# Structural and Functional Characterization of the Gut Microbiota in Elderly Women With Migraine

**DOI:** 10.3389/fcimb.2019.00470

**Published:** 2020-01-29

**Authors:** Juanjuan Chen, Qi Wang, Anqi Wang, Zhanglin Lin

**Affiliations:** ^1^School of Biology and Biological Engineering, South China University of Technology, Guangzhou, China; ^2^BGI-Shenzhen, Shenzhen, China; ^3^BGI Education Center, University of Chinese Academy of Sciences, Shenzhen, China; ^4^School of Future Technology, University of Chinese Academy of Sciences, Beijing, China; ^5^Lanzhou University, Lanzhou, China

**Keywords:** migraine, gut microbiota, elderly women, metagenome-wide association study, structural characterization, functional modules

## Abstract

Migraine is a very common, multifactorial, and recurrent central nervous system disorder that causes throbbing headache, photophobia, phonophobia, nausea, and disability. Migraine occurs more often in females, and its complex physiopathology is not yet fully understood. An increasing number of gastrointestinal disorders have been linked to the occurrence of migraine suggesting that gut microbiota might play a pivotal role in migraine through the gut–brain axis. In the present work, we performed a metagenome-wide association study (MWAS) to determine the relationship between gut microbiota and migraine by analyzing 108 shotgun-sequenced fecal samples obtained from elderly women who suffer from migraine and matched healthy controls. Notably, the alpha diversity was significantly decreased in the migraine group at species, genus, and Kyoto Encyclopedia of Genes and Genomes (KEGG) orthologous levels. Firmicutes, especially the “unfriendly” *Clostridium* spp., were significantly enriched in the migraine group. Conversely, the healthy controls held more beneficial microorganisms, such as *Faecalibacterium prausnitzii, Bifidobacterium adolescentis*, and *Methanobrevibacter smithii*. For functional modules, the migraine group was enriched in gut–brain modules (GBMs) including kynurenine degradation and γ-aminobutyric acid (GABA) synthesis. However, the healthy controls held higher gut metabolic modules (GMMs) including glycolysis, homoacetogenesis, and GBMs including quinolinic acid degradation and *S*-adenosyl methionine (SAM) synthesis. The differences in gut microbiota composition and function between the migraine and healthy groups provided new information as well as novel therapeutic targets and strategies for migraine treatment, which could help to improve the early diagnosis of the disease, as well as the long-term prognosis and the life quality of patients suffering from migraine.

## Introduction

Migraine can cause a severe unilateral throbbing headache or a pulsing sensation, which are typically accompanied by nausea, vomiting, and extreme sensitivity to light and sound, thereby adversely affecting daily activities (MacGregor, 2017). Constipation and mood changes also frequently occur together with migraine (Vacca, 2019). Migraines usually begin in adolescence or early adulthood, it is three times more common in women (17%) than in men (5–8%), and it accounts for over 90% of patients with recurrent headache (MacGregor, 2017). Multiple factors, including inheritance (Ulrich et al., 1999), hormones (Sacco et al., 2012), dietary habits (Nazari and Eghbali, 2012), mental stress (Lipton et al., 2014), and gastrointestinal disorders (Camara-Lemarroy et al., 2016), have been reported as triggers of migraine. For the prognosis, some patients remit, some experience recurrent episodes, and in others the condition evolves into a chronic and more refractory state.

Human gut microbiome, which is considered as the second genome and brain of human body (Li et al., 2014), is thought to be closely related to migraine (Gonzalez et al., 2016). The recent gut–brain axis theory (Carabotti et al., 2015) has proposed a bidirectional communication between the central and enteric nervous systems, linking the emotional and cognitive centers of the brain with the peripheral intestinal functions. Studies have found that irritable bowel syndrome (IBS) occurs in over half of migraine patients (Lau et al., 2014), and inflammatory bowel disease (IBD) patients are 2.7 times more likely to have migraines (Dimitrova et al., 2013). The fact that both IBD and IBS are severe gut disorders associated with gut permeability and inflammation caused by gut microbes through the gut–brain axis (Aggarwal et al., 2012) suggests an important role of gut bacteria in migraine. In an uncontrolled observational study on 1,020 patients, researchers found that multispecies probiotic formulations can reduce the intensity and the frequency of migraine attacks (Straube et al., 2018). However, another randomized placebo-controlled study conducted on 63 patients showed that the use of multispecies probiotics, compared with placebo, does not significantly affect intestinal permeability or inflammation (de Roos et al., 2017). These paradoxical conclusions need further confirmation.

The diagnosis of a migraine is based on signs and symptoms (Bartleson and Cutrer, 2010), such as with or without aura, duration, unilateral, pulsating headache, inability to work, nausea and vomiting, photophobia, and phonophobia. Diagnostic uncertainty has been associated with diagnostic variation (physicians giving different diagnoses to the same patient), over-testing, suboptimal management, more hospitalizations and referrals, and increased health care expenditure. In contrast to traditional pharmacotherapy, which is marked by unavoidable and often dangerous side effects (e.g., analgesics might cause nervous disorders; Do et al., 2019), dietary intervention is thought to be a promising safe therapeutic strategy to prevent migraine through the regulation of gut microbes (Camara-Lemarroy et al., 2016). Both the ketogenic diet (Gross et al., 2019) and the traditional Chinese medicinal plant *Gastrodia elata*, which is also used as food in China (Hua et al., 2019), have been reported to regulate gut microbiota and to promote remission from migraine. The few publications available, which are based on probiotic treatment, however, are with different conclusions about the effects of probiotics on migraine in humans (de Roos et al., 2017; Straube et al., 2018).

In this work, we performed a metagenome-wide association study (MWAS) based on shotgun-sequenced fecal samples obtained from 108 elderly women consisting of 54 migraineurs and 54 healthy subjects in order to shed some light over the connections between gut microbiota composition and function and migraine. The study aimed at discovering differences in gut microbiota that could help to design strategies based on the modulation of the gut microecology that could improve the long-term prognosis of migraine and provide a guidance for early diagnosis and management of this recurrent disease.

## Materials and Methods

### Statement of Human Rights

The data used in this study were obtained from a previous study (Xie et al., 2016) whose ethics statement was approved by the local ethics committee, and an informed consent was collected from each subject. All the procedures followed were in accordance with the ethical standards of the responsible committee on human experimentation (BGI-Shenzhen, China, Ethics Approval No. BGI-IRB 14074).

### Materials

The 108 stool samples, from 54 migraineurs and 54 matched 201 healthy controls, were shotgun sequenced during a former study (Table S1) (Xie et al., 2016). All the samples and clinical indexes were collected by Prof. Spector's group at King's College London. Subjects were excluded if they had a history of chronic serious infection, any current infection, and any type of malignant cancer; individuals who had received antibiotic treatment within 1 month before participating in this study were also excluded.

### Quality Control and Host Genome Filtering

The raw reads that had 50% low-quality bases (quality ≤ 20) or more than five ambiguous bases were excluded. The remaining reads were mapped to the human genome (hg19) by SOAP v2.22 (-m 100 -x 600 -v 7 -p 6 -l 30 -r 1 -M 4 -c 0.95), and the matching reads were removed (Fang et al., 2018). The high-quality non-human reads were defined as clean reads.

### Acquisition of Gene Abundance and Taxonomic Profiles From Metagenomic Samples

The clean reads were aligned against the latest 11.4 M human gut microbial gene catalog (Xie et al., 2016) through SOAP v2.22 (-m 100 -x 600 -v 7 -p 6 -l 30 -r 1 -M 4 -c 0.9) to generate the gene abundance profile. To obtain the taxonomic profiles, metaphlan2 (Truong et al., 2015) (–input_type fastq –ignore_viruses –nproc 6) was used to generate phyla, genera, and species profiles from the clean reads.

### Calculation of Gut Microbiome Functional Profiles

Putative amino acid sequences were translated from the gene catalog (Xie et al., 2016) and aligned against the proteins or domains in the Kyoto Encyclopedia of Genes and Genomes (KEGG) databases (release 79.0, with animal and plant genes removed) using BLASTP (v2.26, default parameter, except -m 8 -e 1e-5 -F -a 6 -b 50). Each protein was assigned to a KEGG orthologous (KO) group on the basis of the highest scoring annotated hit(s) containing at least one segment pair scoring over 60 bits. The relative abundance profile of KOs was determined by summing the relative abundance of genes from each KO using the mapped reads per sample (Xie et al., 2016). The abundance of each gut metabolic module (GMM) (-a 2 -d GMM.v1.07.txt -s average) and gut neuroactive module (GBM) (default parameter) were calculated as shown in the former article (Vieira-Silva et al., 2016; Valles-Colomer et al., 2019).

### Permutational Multivariate Analysis of Variance of the Effect of Related Factors on Gut Microbiome

To evaluate the effects of the clinical and lifestyle factors on the microbiome, we performed the permutational multivariate analysis of variance (PERMANOVA) of the gene abundance of the samples. The Bray–Curtis distance and 9,999 permutations in R (3.2.5, vegan package) were used.

### Richness and Diversity Analysis

Alpha diversity (within samples) at species, genus, and KO levels of the two groups was quantified by the Shannon index on the basis of the relative gene abundance profile.

### Differential Analysis of the Gut Microbiome Between the Two Groups

MWAS was used to investigate the differences in taxon composition between fecal microbiomes of healthy controls and migraineurs. To investigate the specific differences in the gut microbiome composition and function between the migraine group and the healthy controls, first, the top 15 species, the top 10 genera, and the top 5 phyla of each group were selected according to their average relative abundances, and each of the taxon was compared by Wilcoxon rank-sum test to compare their differences between two groups (*p* < 0.05). Second, the significantly different species, genera, phyla (Table S4), GMMs (Table S5), and GBMs (Table S6) between two groups were tested by Wilcoxon rank-sum test (*p* < 0.05). Third, the significantly changed species analyzed above were further analyzed by Spearman's rank correlation (*p* < 0.05) according to their relative abundances in all samples. Then the software Cytoscape 3.4.0 was used to visualize the co-occurrence network of these species.

## Results

### Characterization of the Gut Microbiomes

To investigate the characteristics of gut microbiome in migraine patients, a metagenomic shotgun-sequencing study was performed on a total of 108 fecal samples from 54 individuals with migraine and 54 healthy controls with matched age and body mass index (BMI) (Figure S1). After removal of low-quality and human DNA reads, an average of 7.27 gigabase pairs per sample were aligned to a gut microbiome gene catalog comprising 11.4 million genes (Xie et al., 2016), achieving an average of 77.6 ± 1.6% matched reads per sample (Table S2).

**Figure 1:**
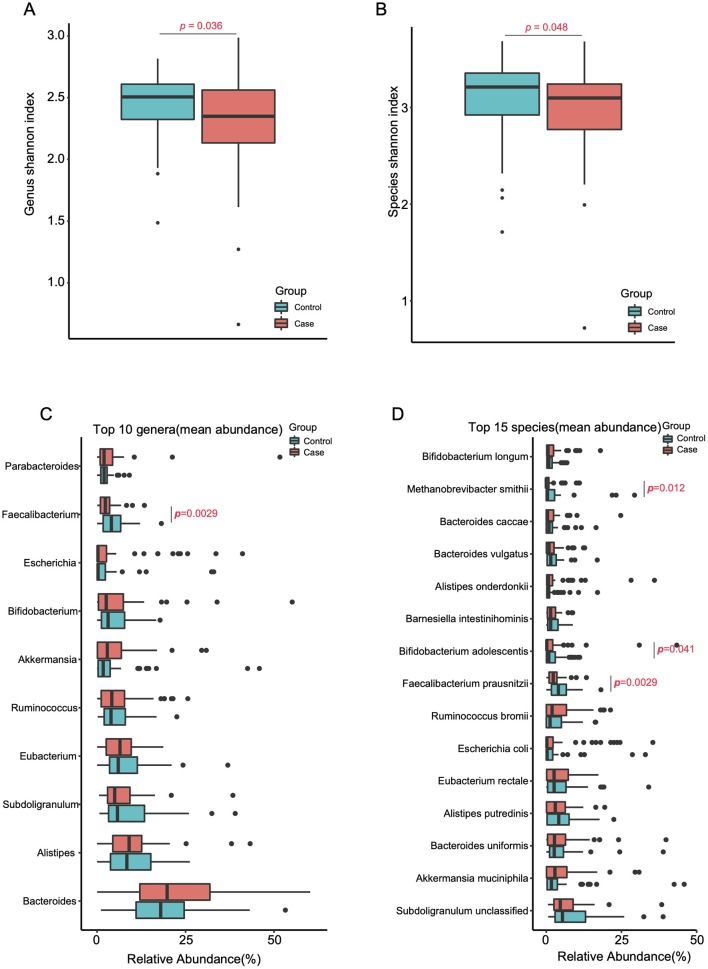
Reduced gut microbial diversity in migraineurs. Alpha-diversity (Shannon index) at the genus **(A)** and species **(B)** levels of the two cohorts (Tested by two-tailed Wilcoxon-rank sum test). The top 10 genera **(C)** and top 15 species **(D)** (mean relative abundance higher than 3.09 and 1.83%, respectively) in the migraine patients and the control individuals (two-tailed Wilcoxon-rank sum test, Tables S4B,C).

PERMANOVA revealed significant differences in the gut microbiome of the two groups (*p* = 0.0066, *R*^2^ = 0.014, Table S1b). The alpha diversity was evidently decreased in the migraine group at both genus (*p* = 0.036, Wilcoxon rank-sum test, Figure 1A) and species (*p* = 0.048, Wilcoxon rank-sum test, Figure 1B) levels, whereas the species richness was not significantly different in the two groups at either level (genus, *p* = 0.64; species, *p* = 1.0; Wilcoxon rank-sum test, Figures S2A,B). Similarly, the KO analysis showed an evident decrease of the alpha diversity in the migraine group (*p* = 0.045, Wilcoxon rank-sum test, Figure S2C), whereas the difference in species richness between the two groups was not significant (*p* = 0.085, Wilcoxon rank-sum test, Figure S2D). The data used in Figure 1 and Figure S1 are included in Table S3.

### Differences of the Gut Microbiome Between Two Groups

To investigate the differences in highly abundant bacteria in the gut of the two groups, we chose the top 15 species, top 10 genera, and top 5 phyla. Interestingly, the migraine group showed significantly higher levels of the phylum Firmicutes (*p* = 0.0023, Figure S3) and a reduction in the level of the beneficial genus *Faecalibacterium* (*p* = 0.0029, Figure 1C) relative to the control group. At the species level (Figure 1D), *Faecalibacterium prausnitzii* (*p*=0.0029), *Bifidobacterium adolescentis* (*p*=0.041), and *Methanobrevibacter smithii* (*p* = 0.012) were significantly enriched in the healthy controls.

**Figure 2:**
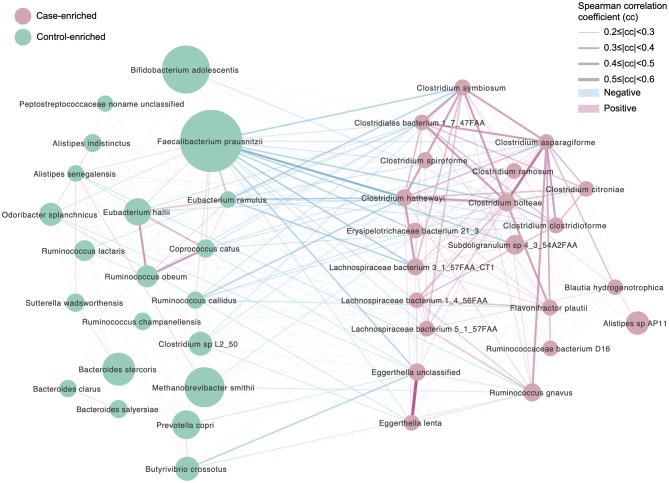
Differentially enriched species, control vs. migraine. The orientation of enrichment was determined by two-tailed Wilcoxon-rank sum test (*U* statistic of two-tail Wilcoxon rank-sum test, *p* < 0.05, FDR< 0.29, Table S4C). The size of the nodes is consistent with the relative abundance of the species. Species were colored according to the extent of enrichment. The edges between the nodes indicate positive (red) and negative (blue) Spearman's correlation, respectively, the line width represents the correlation coefficient calculated for the samples under comparison.

To further illustrate the differences between the two cohorts, 21 and 22 significantly enriched species (Figure 2, *p* < 0.05, false discovery rate [FDR] = 0.14, Table S4) were identified for the migraine group and the healthy controls, respectively. The species enriched in the migraine group were *Blautia hydrogenotrophica, Clostridium asparagiforme, Clostridium clostridioforme, Clostridium bolteae, Clostridium citroniae, Clostridium hathewayi, Clostridium ramosum, Clostridium spiroforme, Clostridium symbiosum, Eggerthella lenta, Flavonifractor plautii, Lachnospiraceae bacterium*, and *Ruminococcus gnavus*. *B. hydrogenotrophica* metabolizes H_2_/CO_2_ to acetate and contributes to the breakdown of plant polysaccharides and proteins in the host (Bernalier et al., 1996). Except for *C. bolteae*, an autism-associated bacterium (Pequegnat et al., 2013), all the other *Clostridium* spp. enriched in the migraine group have been reported to be correlated with solid tissue inflammation (Elsayed and Zhang, 2004b), infection (Finegold et al., 2005), and bacteremia (Elsayed and Zhang, 2004a). *E. lenta* and *F. plautii* can cause bacteremia (Wong et al., 2014) and bloodstream infection (Berger et al., 2018), respectively. *R. gnavus*, which degrades mucin (Crost et al., 2016), is enriched in IBD patients (Hall et al., 2017) and can cause bacteremia (Hansen et al., 2013).

The species significantly enriched in the controls were *Bacteroides clarus, Bacteroides intestinalis, Bacteroides salyersiae, Bacteroides stercoris, Butyrivibrio crossotus, Clostridium* sp. L2_50, *Coprococcus catus, Eubacterium hallii, Eubacterium ramulus, Odoribacter splanchnicus, Peptostreptococcaceae noname* unclassified, *Prevotella copri, Ruminococcus callidus, Ruminococcus champanellensis, Ruminococcus obeum*, and *Sutterella wadsworthensis*. In this list, at least one bacterium regarded as “unfriendly” can be found: *O. splanchnicus*, which has been isolated from the crevicular spaces of dogs with periodontitis (Hardham et al., 2008). However, many more species regarded as beneficial were found in the healthy subjects compared with the migraineurs. *B. clarus* is significantly decreased in colorectal cancer (Watanabe et al., 2010; Liang et al., 2017), and its enrichment in controls may be beneficial. *S. wadsworthensis* protects against IBD (Wexler et al., 1996); *C. catus* ferments fructose, lactate, and pyruvate to short-chain fatty acids (SCFAs) (Holdeman and Moore, 1974); *E. hallii* contributes to the intestinal propionate formation (Engels et al., 2016) and improves insulin sensitivity (Udayappan et al., 2016); *E. ramulus* produces propionic acid and dihydroxyphenylacetic acid (Schneider and Blaut, 2000; Braune et al., 2001); *R. obeum* plays an important role in the recovery process from *Vibrio cholerae* infection (Lawson and Finegold, 2015). In addition, species involved in polysaccharide degradation were also enriched in the healthy controls. These were *B. intestinalis*, which degrades arabinoxylan for energy acquisition (Wang et al., 2016); *B. crossotus*, which metabolizes polysaccharides into simpler sugars (Kelly et al., 2010); and *R. callidus* and *R. champanellensis*, which degrade various plant hemicelluloses and cellulose (Chassard et al., 2012).

### Functional Alterations in the Gut Microbiome of Migraineurs

**Figure 3:**
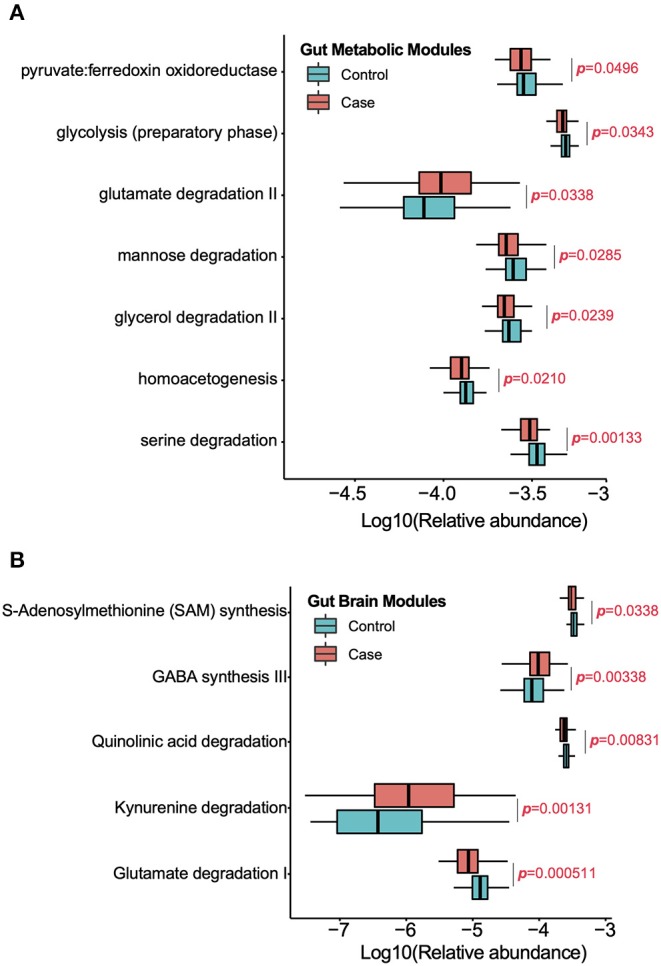
Significantly enriched GMMs **(A)** and GBMs **(B)** in each group were analyzed by Wilcoxon rank sum test (*p* < 0.05). The Y-axis is the significantly enriched GMMs and GBMs in two groups and X-axis is the logarithm of relative abundance for each GMM and GBM. *P*-value was labeled.

Significantly different GMMs and GBMs between two groups were analyzed (*p* < 0.05, Wilcoxon rank-sum test). Seven significantly changed GMMs between two groups were observed (Figure 3A), and *glutamate degradation II* is the only GMM that is significantly higher in the migraine group. *Serine degradation, homoacetogenesis, glycerol degradation, mannose degradation, glycolysis (preparatory phase)*, and *pyruvate:ferredoxin oxidoreductase* were the six GMMs found to be enriched in the healthy controls. *Serine* is a major energy and SCFAs contributor in the human body and can be degraded to pyruvate by *Escherichia coli* (Su et al., 1989). *Homoacetogenesis* produces acetate by consuming hydrogen (Ni et al., 2011). Higher *glycerol degradation* can reduce the *triglycerides synthesis* and *phospholipids synthesis*. *Mannose*, which has been reported to impair tumor growth and to enhance chemotherapy, can be degraded into fructose, which is then catabolized to lactate, a precursor of SCFAs (Gonzalez et al., 2018). *Glycolysis (preparatory phase)* consumes energy to convert glucose into two 3-carbon molecules (Kathagen et al., 2013). *Pyruvate:ferredoxin oxidoreductase* is a key enzyme in metabolism that catalyzes pyruvate to acetyl-CoA and CO_2_ (Furdui and Ragsdale, 2000). Taken together, these results indicated that the gut microbiota of the healthy controls were more active in energy metabolism and SCFA synthesis, which might be beneficial in maintaining their health.

Five significantly changed GBMs between two groups were observed (Figure 3B). *Glutamate degradation I, quinolinic acid degradation*, and *S-adenosyl methionine* (SAM) *synthesis* were predominant in the control group, which meant that there was a decrease in the levels of glutamate and quinolinic acid while an increase in those of the SAM in the healthy controls. Glutamate is a neurotransmitter in the healthy brain. Quinolinic acid is an endogenous *N*-methyl-d-aspartate (NMDA) receptor agonist and possesses neuroactive activity (Heyes et al., 1992). SAM is a major methyl donor in the brain. Conversely, *kynurenine degradation* and γ*-aminobutyric acid (GABA) synthesis III* were enriched in the migraine group. Kynurenine is a metabolite of tryptophan and can be degraded to quinolinic acid and kynurenic acid, an NMDA antagonist, and is thought to be involved in the pathophysiology and pathogenesis of schizophrenia. GABA is the chief inhibitory neurotransmitter in the developmentally mature mammalian central nervous system.

## Discussion

Migraine susceptibility is multifactorial with genetic, hormonal, and environmental factors. The physiopathology of migraine is complex and still not fully understood. Recent reports demonstrate an increased frequency of gastrointestinal disorders, such as *Helicobacter pylori* infection, IBS, gastroparesis, hepatobiliary disorders, celiac disease, and alterations in the microbiota have been linked to the occurrence of migraine (Camara-Lemarroy et al., 2016). However, several case–control studies based on probiotic treatment have different conclusions (de Roos et al., 2017; Straube et al., 2018). Up to now, the precise characteristics and changes of gut microbiome in migraine were not fully elucidated. In this study, we presented the first set of evidence obtained from human cohorts for a significant gut microbiome dysbiosis from both compositional and functional aspects in migraine.

Significant differences in the gut microbiota composition were observed in our study. First, the migraine group showed a significantly lower alpha diversity at species, genus, and KO levels than did the healthy controls, which might be caused by a significant depletion in some highly abundant bacteria in migraine, such as *Faecalibacterium, F. prausnitzii, B. adolescentis*, and *M. smithii*. *F. prausnitzii* is a major producer of butyrate (Machiels et al., 2014), *B. adolescentis* exhibits strain-specific effects in the alleviation of constipation (Wang et al., 2017), and *M. smithii*, a predominant archaeon in the human gut, can affect the specificity and efficiency of dietary polysaccharides to influence host calorie harvest and adiposity (Samuel et al., 2007). Notably, Firmicutes, the main bacterial phylum in the gut, was significantly enriched in migraine. Second, some species thought to be detrimental to human health, especially *Clostridium* spp., were significantly enriched in migraineurs. Third, the controls held more beneficial microorganisms, such as *B. adolescentis, F. prausnitzii*, and *Bacteroides intestinalis*, suggesting that the control subjects held a healthier gut microenvironment than did migraineurs. Notably, some “unfriendly” species, such as *Odoribacter splanchnicus* and *Prevotella copri*, were also elevated in controls, suggesting the likelihood of susceptibility to intestinal inflammation (Hardham et al., 2008) and arthritis (Scher et al., 2013) in the seemingly healthy control subjects.

From a functional point of view, significant changes in GMMs and GBMs between two groups were observed. Interestingly, the migraineurs had less GMMs and GBMs enriched than did the controls. The healthy controls held more modules related to substrates metabolism, glycolysis (preparatory phase), and SCFA production, whereas *glutamate degradation II* is the only significantly changed GMM enriched in migraine. These results suggested that migraineurs might suffer from metabolic dysfunctions and insufficient SCFA synthesis.

For GBMs, *kynurenine degradation* and *GABA synthesis III* were significantly higher in the migraine group. The higher *kynurenine degradation* module observed in the migraine group suggested the potential presence of elevated concentration of its catabolites, that is, the neuroexcitatory quinolinic acid and neuroinhibitory kynurenic acid, which can cause diseases in the nervous and immune systems (Heyes et al., 1992). The higher GABA synthesis observed in the migraine group indicated the potential presence of higher levels of GABA in the brain, which might be beneficial for health. Oral GABA administration was reported to relieve anxiety (Abdou et al., 2006), improve mood (Sakashita et al., 2019), and reduce symptoms of premenstrual syndrome (Rapkin and Akopians, 2012). In addition, GABA also supports the physiologic adjustment of pituitary gland function and controls growth hormone secretion from the pituitary gland (Acs et al., 1987), promotes muscle protein synthesis (Olarescu et al., 2000), stabilizes blood pressure (Ma et al., 2015), and relieves pain (Jasmin et al., 2004). On the contrary, the higher capability of *glutamate degradation I* was observed in the healthy group. Glutamate is the principal excitatory neurotransmitter in the healthy brain and provides energy for normal brain function, whose depletion is a common feature of many neuropsychiatric conditions such as schizophrenia (Zhou and Danbolt, 2014). In addition, higher quinolinic acid degradation was observed in the control group. Quinolinic acid is an NMDA antagonist, has a potent neurotoxic effect, and may be involved in many psychiatric disorders and neurodegenerative processes in the brain. These GBM changes reminded us that the healthy volunteers might also have a risk in getting mental disorders, although they have no significant signs. Higher synthesis of SAM in controls indicated higher levels of SAM, an antidepressant with few side effects (Young and Shalchi, 2005). The functional redundancy, which was reported by Kang et al. (2015), was also found in both migraine group and healthy controls in our study, which meant that the gut microbiome can self-regulate to relieve symptoms when their hosts suffered from migraine. In addition, changes in some unhealthy gut microbiome composition and function in the healthy group might also indicate that migraine may still happen in seemingly healthy people in the future.

In summary, our results revealed a significant decrease in species diversity and metabolic functions in the gut microbiota of migraine sufferers, which highlighted the importance of maintaining species diversity to improve the gut microecosystem stability. Additionally, the monitoring of harmful bacteria such as *Clostridium* spp. could be a new strategy for early diagnosis and timely prevention of migraine. In addition, proper probiotics could be supplemented to migraineurs to treat their intestinal dysbiosis or prevent them from gut disorders, which may reduce the occurrence of migraine attacks. Our findings revealed that gut microbiota can be a potential target for migraine management and offered not only novel promising treatment strategies but also an important functional basis for future research on this disease.

## Data Availability Statement

The datasets analyzed in this study can be found in the European Bioinformatics Institute (EBI) with the accession ID ERP010708 or in the China National Genebank (CNGB) with a program ID CNPhis0000107.

## Ethics Statement

The studies involving human participants were reviewed and approved by Research Health Authority NRES Committee London - Westminster. The patients/participants provided their written informed consent to participate in this study.

## Author Contributions

QW and JC conceived and designed the study. QW and AW were in charge of data analysis and graphics presentation. JC wrote the paper. QW and ZL reviewed and revised the manuscript. All authors read and approved the manuscript.

### Conflict of Interest

JC and QW are/were employed by BGI-Shenzhen. The remaining authors declare that the research was conducted in the absence of any commercial or financial relationships that could be construed as a potential conflict of interest.
