# Supplementary material for: Structural and Functional Characterization of the Gut Microbiota in Elderly Women With Migraine
Source: Front Cell Infect Microbiol. 2020 Jan 29;9:470. doi: 10.3389/fcimb.2019.00470 (PMC7001586; doi:10.3389/fcimb.2019.00470)
Supplement: Supplementary file 7 [file Data_Sheet_1.docx]

Supplementary Material

# Supplementary Figures and Tables

## Supplementary Figures

**Supplementary Figure 1.** Age and body mass index showed no significant difference between two groups.

**Supplementary Figure 2.** Richness and alpha diversity in migraine. No significant changes for gut microbial richness at the genus (a) and species (b) levels. Significant KO diversity by Shannon index (c) and non-significant KO richness (b) (Test by two-tailed Wilcoxon-rank sum test). Box plots showing both the richness values or diversity values and their density.

**Supplementary Figure 3.** The top 5 phyla (the mean relative abundance more than 5.63%) in the cohort between migraine patients and control individuals (two-tailed Wilcoxon-rank sum test, Table S4a).

# 1.2 Supplementary Tables

Table S1. Clinical parameters of the samples.

Table S2. The sequencing information of the sample.

Table S3. The alpha diversity data.

Table S4. The Metaphlan2 results.

Table S5. Differentially enriched gut metabolic modules (GMMs, Table S5a) and gut neuroactive modules (GNMs, Table S5b): The relative abundance and statistical test of the GMMs and GNMs.
